# Oral pilocarpine for the treatment of dry eye in patients with Sjögren’s syndrome

**DOI:** 10.5935/0004-2749.20220069

**Published:** 2022

**Authors:** Sergio Felberg, Paulo Elias Correa Dantas, Elcio Hideo Sato

**Affiliations:** 1 Department of Ophthalmology, Irmandade da Santa Casa de Misericórdia de São Paulo, São Paulo, SP, Brazil; 2 Department of Ophthalmology and Visual Science, Escola Paulista de Medicina, Universidade Federal de São Paulo, São Paulo, SP, Brazil

**Keywords:** Dry eye, Pilocarpine/therapeutic use, Sjögren’s syndrome/drug therapy, Tears, Olho seco, Pilocarpina/uso terapêutico, Síndrome de Sjögren/tratamento farmacológico, Lágrimas

## Abstract

**Purpose:**

To evaluate the efficacy of oral pilocarpine (20 mg daily) for the treatment of dry eye in patients with Sjogren’s Syndrome. The frequency of side effects reported during the treatment was also investigated.

**Methods:**

In this placebo-controlled crossover study, 32 patients with Sjögren’s syndrome were enrolled to receive either oral pilocarpine or placebo for 10 weeks. Following a 2-week washout period, the treatment was inverted for each patient for the same duration. Assessments included the quality of life National Eye Institute Visual Function Questionnaire-25 (NEI-VFQ-25), dry eye specific questionnaire Ocular Surface Disease Index, non-invasive breakup time, invasive breakup time with fluorescein, corneal and conjunctival staining patterns with the use of fluorescein and rose bengal staining, Schirmer’s test, and tear ferning test.

**Results:**

According to the NEI-VFQ-25, there was statistically significant improvement in the quality of life following oral pilocarpine. Similar results were observed for ocular discomfort, as determined by the Ocular Surface Disease Index. All clinical tests showed favorable and statistically significant results following the use of oral pilocarpine. Regarding the analysis of tear samples, there was an improvement in the quality of tear film. This was evidenced by the modification of the patterns observed in the tear ferning test. Side effects were reported by 96.8% and 56.2% of the patients who received pilocarpine and placebo, respectively. Sweating was the most frequently reported side effect (67.74% *versus* 11.11%, respectively).

**Conclusions:**

Although the treatment was associated with a high frequency of side effects, oral pilocarpine (20 mg daily) was able to relieve discomfort related to dry eyes in patients with Sjögren’s syndrome and induce favorable structural changes in the tear film.

**Clinical Trial registration on ClinicalTrials.gov:** NCT04470479

## Abstract (pt)

**Objetivos:**

Avaliar o alívio de sintomas e sinais relacionados à secura ocular e na qualidade de vida de pacientes com síndrome de Sjögren tratados com o uso oral de pilocarpina na dose diária de 20mg. A frequência dos efeitos colaterais relatados com o tratamento também foi estudada.

**Métodos:**

Trata-se de estudo cruzado e placebo-controlado, que envolveu 32 pacientes com síndrome de Sjögren em uso de pilocarpina oral ou placebo, por dez semanas. Após duas semanas sem medicações, houve a inversão dos tratamentos para cada paciente, por mais dez semanas. As avaliações foram feitas por meio do questionário de qualidade de vida NEI-VFQ-25, questionário olho seco específico *Ocular Surface Disease Index*, tempo de ruptura do filme lacrimal não invasivo, tempo de ruptura do filme lacrimal com fluoresceína, avaliação da superfície ocular com os corantes fluoresceína e rosa Bengala, teste de Schirmer e teste de cristalização do filme lacrimal.

**Resultados:**

Houve melhora estatisticamente significante na qualidade de vida medida pelo questionário NEI-VFQ-25 e no desconforto ocular avaliado pelo *Ocular Surface Disease Index*, após o tratamento. Todos os testes clínicos sofreram influência favorável e estatisticamente significante durante a fase de tratamento com pilocarpina oral. Em relação à análise de amostras de lágrimas, ocorreram alterações estruturais indicando melhora no padrão de cristalização do filme lacrimal. Os efeitos colaterais com o uso de pilocarpina foram relatados por 96,8% dos pacientes com a pilocarpina e 56,2% com placebo. Sudorese foi o efeito colateral mais frequentemente relatado (pilocarpina = 67,74%; placebo = 11,11%).

**Conclusões:**

O uso oral de pilocarpina na dose diária de 20mg foi capaz de aliviar as queixas de desconforto relacionadas ao ressecamento ocular em pacientes com síndrome de Sjögren, produzir impacto positivo na qualidade de vida dos pacientes e induzir mudanças estruturais favoráveis no filme lacrimal, embora os efeitos colaterais relatados tenham ocorrido com alta frequência.

## INTRODUCTION

Sjögren’s syndrome (SS) is a systemic disorder with chronic evolution and multifactorial etiology, involving the immune system and production of autoantibodies. It is characterized by progressive lympho-plasmatic infiltration of the exocrine glands, mainly the salivary, and lacrimal glands. Consequently, this results in the replacement of acinar tissue by fibrous material, leading to the development of clinical symptoms such as xerostomia and ocular dryness^(1,2)^. Different therapeutic modalities have been proposed to relieve patients’ symptoms and modify the course of the disease^(3)^. Among them, the most frequently utilized treatment for dry eye is therapy replacement, which includes the use of topical artificial lubricants as substitutes for the naturally produced tear. Several formulations are commercially available; however, none of those exhibit identical characteristics to the complex structure of the natural tear film^(4)^. For this reason, previous studies assessed the possibility to stimulate tear production using formulations administered by topical or systemic routes, and their effects on ocular dryness^(5,6)^. The most studied oral drug for this purpose is pilocarpine hydrochloride, a parasympathomimetic cholinergic drug with affinity to muscarinic receptors^(7)^. Nevertheless, the usefulness of this treatment for the relief of signs and symptoms in patients with dry eyes remains uncertain.

The purpose of the present study was to evaluate the efficacy of oral pilocarpine (20 mg daily) for the treatment of dry eye and its effect on the quality of life of patients with SS. The tear ferning test was performed using tear samples to detect possible structural changes in the tear film induced by oral pilocarpine. The frequency of systemic side effects was also observed.

## METHODS

This was a prospective, placebo-controlled, crossover study in which patients with SS were allocated according to a computer-generated schedule to receive pilocarpine hydrochloride (5 mg) or placebo four times daily for a period of 10 weeks. After a 2-week washout period, a mandatory inversion of the treatment was performed for an additional 10 weeks.

The study group was composed of patients with primary or secondary SS from the Ocular Surface and Tear Ambulatory of the Corneal Sector of the Department of Ophthalmology of Santa Casa de São Paulo (São Paulo, Brazil).

### Inclusion criteria

- Patients with SS according to the criteria defined by the American-European Consensus for the diagnosis of SS^(8)^.
- Controlled collagen disease prior to the initiation of the trial.
- Any systemic therapy should have been instituted at least 2 months prior to the initiation of the trial.
- Age ≥18 years.

### Exclusion criteria

- Ocular surface diseases or eyelid abnormalities not related to SS.
- Temporary or permanent punctal occlusion.
- Usage of contact lenses.
- Usage of systemic medication known to influence tear flow.
- Necessity to modify the systemic treatment of previous diseases during the trial.
- Pregnancy or breastfeeding.
- Known hypersensitivity to pilocarpine hydrochloride.
- Severe cardio-pulmonary disease.

Patients who required topical use of eye drops other than lubricants and those who inappropriately used the provided tablets were excluded from the final analysis.

### Assessments

Patient assessments were carried out prior to the initiation of the trial, 10 weeks later, and at the end of the trial (week 22) following treatment inversion. The use of ocular lubricants was discontinued ≥2 h before the assessments.

The validated questionnaire NEI-VFQ 25 was applied to assess the impact of dry eye on the quality of life of each patient. A global index was generated ranging 0-100; higher scores indicated lower negative impact of the disease in the quality of life^(9)^. The dry eye specific questionnaire Ocular Surface Disease Index (OSDI^®^; Allergan, Irvine, CA, USA) was applied to assess the impact of dry eye on ocular discomfort. The survey generated a score ranging 0-100 (0 indicated absence of eye discomfort and 100 represented maximum eye discomfort)^(10)^. The evaporative dry eye component of both eyes was studied through the non-invasive measurement of the tear breakup time using the *Tearscope-plus*^®^ device (Keller, England, Inc) and fluorescein 1% staining. Next, the impact of dryness on the ocular surface was investigated with fluorescein and rose bengal vital dyes. A corneal staining score was generated from the fluorescein staining (0 = absence of keratitis; 1 = mild keratitis with distant staining points from each other; 2 = moderate keratitis with staining points closer; 3 = severe keratitis with confluent staining points). Subsequently, the tear flow was assessed by Schirmer’s test, performed without using anesthetics (Schirmer’s test I). Finally, the traditional van Bijesterveld score was calculated following the evaluation of the ocular surface with rose bengal 1% staining (scores: 0-9)^(11)^. The entire process was performed and evaluated by a single examiner. The following day, the quality of the tear film was studied using the tear ferning test using collected tear samples, according to the technique proposed by Rolando (scores I, II, III and IV, according to the appearance of the ferning branches observed). Scores I and II are traditionally considered normal, whereas scores III and IV are considered abnormal)^(12)^. Only samples from the right eye of each patient were collected. The ferning patterns were evaluated and classified by two independent observers. The frequency of side effects at the end of each phase was recorded. A questionnaire containing a list of the most frequent side effects reported after the use of oral pilocarpine was distributed to the patients.

We compared the means of the variables under the effect of pilocarpine and placebo in relation to baseline. Student’s t-test was applied for paired samples, while the Wilcoxon test was utilized for quantitative and qualitative data analysis. For the tear ferning test, the Kappa coefficient was calculated to assess inter-observer reproducibility. The statistical level of significance was set at 5%.

## RESULTS

Thirty-two patients (31 females, one male) completed the protocol in agreement with all requirements. Fourteen and 18 patients had primary and secondary SS, respectively. The patients’ age ranged 27-69 years (mean ± standard deviation: 52.1 ± 10.59 years).

Table 1 shows the comparison results of the global indexes observed obtained using the NEI-VFQ-25 questionnaire. Compared with baseline, statistically significant difference was noted after the use of pilocarpine; however, the difference recorded after using the placebo was not significant. Similarly, the global score generated by the OSDI questionnaire demonstrated a favorable and statistically significant variation only during the phase in which pilocarpine was administered (Table 2). Both, the non-invasive breakup time and traditional breakup time assessments exhibited statistically significant variations compared with the values observed at baseline (Table 3). Significant improvement in the ocular surface was observed, as assessed by fluorescein and rose bengal staining (Tables 4 and 5). Notably, complete normalization of the ocular surface was not reached, since the damage persisted in most patients. Concerning the tear flow assessed with the Schirmer’s test, an increase in tearing in both eyes was observed after the use of the drug (p<0.001), but not after placebo (Table 6). Regarding the tear ferning test, for both observers, there was a significant improvement in the patterns of the tear samples after using pilocarpine compared with baseline (p<0.001); however, this effect was absent after use of placebo (Table 7). The reproducibility of the evaluations between the two examiners was considered moderate (Kappa coefficient: baseline = 0.51; after pilocarpine = 0.43; after placebo = 0.41). Concerning the systemic effects reported by patients after using pilocarpine, only one patient (3.2%) did not report any side effect. The remaining 31 patients (96.8%) had at least one adverse effect associated with the drug; sweating was the most frequently reported side effect (pilocarpine = 67.74% *versus* placebo = 11.11%). Eighteen patients (56.2%) experienced at least one side effect after taking placebo. Table 8 shows the frequency of side effect occurrence after receiving pilocarpine and placebo.

**Table 1:** Comparative results obtained using the quality of life questionnaire NEI-VFQ-25

NEI-VFQ-25 (phase)	Mean	SD	Range	p-value^*^
T0	45.92	17.68	16.96-84.12	
Pilocarpine	56.74	17.51	21.58-87.16	
Placebo	46.87	19.47	16.78-85.79	
Pilocarpine-T0	10.82	12.46		<0.001
Placebo-T0	0.96	8.57		0.532
Pilocarpine-Placebo	9.87	10.53		<0.001

* p<0.05 denotes statistically significant difference (Student’s t-test).

NEI-VFQ-25= National Eye Institute Visual Function Questionnaire-25; Pilocarpine-Placebo= pilocarpine in relation to placebo; Pilocarpine-T0, pilocarpine in relation to baseline; Placebo-T0, placebo in relation to baseline; SD= standard deviation; T0= baseline.

**Table 2:** Comparative results obtained using the dry eye questionnaire OSDI

OSDI (phase)	Mean	SD	Range	p-value^*^
T0	49.61	14.69	20.45-81.81	
Pilocarpine	39.62	16.06	13.63-82.50	
Placebo	52.25	12.48	27.50-77.08	
Pilocarpine-T0	-9.99	14.57		0.001
Placebo-T0	2.64	9.38		0.122
Pilocarpine-Placebo	-12.63	13.33		0.000

* p < 0.05 denotes statistically significant difference (Student’s t-test).

OSDI= Ocular Surface Disease Index; Pilocarpine-Placebo= pilocarpine in relation to placebo; Pilocarpine-T0= pilocarpine in relation to baseline; Placebo-T0= placebo in relation to baseline; SD= standard deviation; T0= baseline.

**Table 3:** Comparative results obtained using the non-invasive breakup time and breakup time with fluorescein

	Nl-BUT (phase)	Mean time (s)	SD	Range	p-value^*^
RE	T0	4.76	2.54	1.4-9.4	
	Pilocarpine	6.23	3.32	1.3-12.4	
	Placebo	5.15	2.48	1.6-9.4	
LE	TO	5.04	1.87	1.6-9.4	
	Pilocarpine	7.02	2.62	1.7-12.7	
	Placebo	5.42	2.57	1.8-12.4	
RE	Pilocarpine-T0	1.47	1.50		<0.001
	Placebo-T0	0.38	1.46		0.147
	Pilocarpine-Placebo	1.08	1.99		0.004
LE	Pilocarpine-T0	1.97	1.23		<0.001
	Placebo-T0	0.38	1.36		0.126
	Pilocarpine-Placebo	1.59	1.78		<0.001
	**BUT (phase)**	**Mean time (s)**	**SD**	**Range**	p-value^*^
RE	TO	3.38	1.36	1.3-5.6	
	Pilocarpine	6.60	2.73	1.1-10.2	
	Placebo	3.91	1.46	1.4-6.8	
LE	TO	3.74	1.19	1.6-5.4	
	Pilocarpine	6.62	2.48	1.4-9.5	
	Placebo	3.76	1.36	1.3-6.5	
RE	Pilocarpine-T0	3.23	1.69		<0.001
	Placebo-T0	0.54	1.15		0.013
	Pilocarpine-Placebo	2.69	1.95		<0.001
LE	Pilocarpine-T0	2.88	1.67		<0.001
	Placebo-T0	0.02	1.21		0.930
	Pilocarpine-Placebo	2.86	1.89		<0.001

* p<0.05 denotes statistically significant difference (Student’s t-test).

BUT= breakup time with fluorescein; LE= left eye; NI-BUT, non-invasive breakup time; Pilocarpine-Placebo, pilocarpine in relation to placebo; Pilocarpine-T0, pilocarpine in relation to baseline; Placebo-T0= placebo in relation to baseline; RE= right eye; SD= standard deviation; T0= baseline.

**Table 4:** Comparative results of corneal staining obtained using fluorescein

	Corneal staining (phase)	Mean score	SD	Range	p-value^*^
RE	T0	2.19	0.93	0-3	
	Pilocarpine	1.41	1.01	0-3	
	Placebo	2.22	0.79	0-3	
LE	T0	2.34	0.82	0-3	
	Pilocarpine	1.41	1.04	0-3	
	Placebo	2.25	0.91	0-3	
RE	Pilocarpine-T0	-0.78	1.04		0.001
	Placebo-T0	0.03	0.74		1.000
LE	Pilocarpine-T0	-0.94	0.95		<0.001
	Placebo-T0	-0.09	0.96		0.697

* p<0.05 denotes statistically significant difference (Wilcoxon test).

LE= left eye; Pilocarpine-T0= pilocarpine in relation to baseline; Placebo-T0= placebo in relation to baseline; RE= right eye; SD= standard deviation; T0= baseline.

**Table 5:** Comparative results for the van Bijesterveld ocular surface score obtained using rose bengal

	Rose bengal (phase)	Mean score	SD	Range	p-value^*^
RE	T0	6.84	2.09	2-9	
	Pilocarpine	4.25	2.72	0-8	
	Placebo	6.88	2.02	1-9	
LE	T0	6.78	1.91	2-9	
	Pilocarpine	4.41	2.73	0-9	
	Placebo	6.66	2.04	1-9	
RE	Pilocarpine-T0	-2.59	1.92		<0.001
	Placebo-T0	0.03	1.67		0.939
LE	Pilocarpine-T0	-2.38	1.68		<0.001
	Placebo-T0	-0.13	1.10		0.623

* p<0.05 denotes statistically significant difference (Wilcoxon test).

LE= left eye; Pilocarpine-T0= pilocarpine in relation to baseline; Placebo-T0= placebo in relation to baseline; RE= right eye; SD= standard deviation; T0= baseline.

**Table 6:** Comparative results obtained using the Schirmer’s test I

	Schirmer’s test (phase)	Mean (mm)	SD	Range	p-value^*^
RE	T0	3.47	2.14	0-7	
	Pilocarpine	4.72	3.31	0-11	
	Placebo	3.25	2.37	0-10	
LE	T0	3.31	2.02	0-7	
	Pilocarpine	4.81	3.19	0-12	
	Placebo	3.28	2.05	0-8	
RE	Pilocarpine-T0	1.25	1.98		0.001
	Placebo-T0	-0.22	1.41		0.386
	Pilocarpine-Placebo	1.47	2.55		0.003
LE	Pilocarpine-T0	1.50	1.68		<0.001
	Placebo-T0	-0.03	1.20		0.884
	Pilocarpine-Placebo	1.53	2.12		<0.001

* p<0.05 denotes statistically significant difference (Student’s t-test).

LE= left eye; Pilocarpine-Placebo= pilocarpine in relation to placebo; Pilocarpine-T0= pilocarpine in relation to baseline; Placebo-T0= placebo in relation to baseline; RE= right eye; SD= standard deviation; T0= baseline.

**Table 7:** Comparative results obtained using the tear ferning test according to Rolando’s score

	TFT (phase)	Mean score	SD	Range	p-value^*^
RE (observer 1)	T0	3.38	0.71	2-4	
	Pilocarpine	2.19	1.23	1-4	
	Placebo	3.28	0.92	1-4	
RE (observer 2)	T0	3.47	0.62	2-4	
	Pilocarpine	2.50	1.02	1-4	
	Placebo	3.47	0.76	1-4	
RE (observer 1)	Pilocarpine-T0	-1.19	1.06		<0.001
	Placebo-T0	-0.97	0.82		0.433
RE (observer 2)	Pilocarpine-T0	-0.97	0.90		<0.001
	Placebo-T0	0.00	0.80		1.000

* p<0.05 denotes statistically significant difference (Wilcoxon test).

Pilocarpine-T0= pilocarpine in relation to baseline; Placebo-T0= placebo in relation to baseline; RE= right eye; SD= standard deviation; T0= baseline; TFT= tear ferning test.

**Table 8:** Frequency of side effects when using pilocarpine and placebo

Pilocarpine			Placebo		
**Side effect**	**N**	**%**	**Side effect**	**N**	**%**
Sweating	21	67.74	Abdominal pain	6	33.33
Salivation	16	51.61	Weakness	4	22.22
Chills	8	25.81	Blurred vision	4	22.22
Nausea	7	22.58	Nausea	3	16.67
Abdominal pain	7	22.58	Dizziness	3	16.67
Diuresis	6	19.35	Sweating	2	11.11
Dizziness	6	19.35	Diuresis	2	11.11
Flush	5	16.13	Flush	2	11.11
Rhinitis	5	16.13	Rhinitis	2	11.11
Weakness	5	16.13	Diarrhea	2	11.11
Blurred vision	5	16.13	Shaking hand	2	11.11
Shaking hand	4	12.90	Chills	1	5.56
Tachycardia	3	9.68	Salivation	1	5.56
Diarrhea	2	6.45	Tachycardia	1	5.56
Headache	2	6.45	Headache	1	5.56

N denotes the number of patients who experienced a certain adverse effect within the group of patients who experienced side effects. Percentages were calculated based on 31 and 18 patients in the pilocarpine and placebo groups, respectively, who had at least one adverse effect.

## DISCUSSION

The results of this study reveal that the systemic use of pilocarpine at a daily dose of 20 mg induced quantitative and qualitative changes in the tear film in patients with SS. These changes were possibly reflected in the relief of symptoms and signs related to ocular dryness and improvement of patients’ quality of life. Clinical trials that aim to evaluate the effectiveness of a certain treatment in the management of dry eye tend to have several limitations. These limitations can compromise the evidence of these relationships and, consequently, the study conclusions. Factors (e.g., the environment, dietary habits, and medications that cannot be discontinued for ethical or safety reasons), as well as the specific characteristics of the disease in each patient, can directly impact the observed results. Patients with autoimmune diseases, such as SS, are characterized by heterogeneity in their clinical features. Therefore, crossover studies are the most appropriate design for clinical trials involving patients with autoimmune dry eye, offering them the opportunity to receive both treatments.

For patients who initiated the trial using pilocarpine, the 2-week washout period ensures the absence of any residual effects that could have influenced the data collected during the placebo phase. Pilocarpine has a very short half-life and a maximum period of action of 6 h after ingestion and rapid elimination. Hence, theoretically there is no residual pilocarpine found in the peripheral blood 24 h after its use^(7)^. In the present study, symptoms related to dry eye (verified by the OSDI questionnaire) demonstrated a beneficial effect with the use of medication. The variation in the score provided by the OSDI was statistically significant after pilocarpine (49.61 ± 14.69 before the initiation of the trial and 39.62 ± 16.06 after drug use). However, the mean values did not reach those traditionally observed in patients without dry eye (scores ranging 0-12)^(10)^. This suggests that, despite relief, the patients remained symptomatic. This relief may have been responsible for the improvement in quality of life determined using the NEI-VFQ-25 questionnaire. Other trials have tested pilocarpine dosages ranging 5-30 mg on a daily basis. The findings verified that the observed therapeutic effects and frequency of side effects were dose dependent^(13-16)^. A study showed that 9 mg of oral pilocarpine daily for 1 month was sufficient to induce subjective improvement of dry eye sensation in 26% of the patients with SS^(17)^. Another study revealed that an increase in pilocarpine dose from 20 mg to 30 mg daily, resulted in significant improvements in six of the eight items in a questionnaire related to dry eyes symptoms. When the lowest dose was used, improvement was observed only in three items^(15)^. Another study detected subjective improvement of dry eye symptoms, measured using a visual analog scale, in all 30 patients with SS included in the trial with either 20 mg or 30 mg of pilocarpine daily; the effect was more significant in those who received the highest dose^(14)^.

Regarding the production of tears verified by the Schirmer’s test, the present study revealed a statistically significant increase in mean values after the use of pilocarpine. Similar findings were reported by Solans et al. following the administration of 20 mg of oral pilocarpine daily for 6 months^(18)^. A previous study investigating the effects of 10 mg of pilocarpine daily in patients with SS found that this dose was not sufficient to induce changes after 12 weeks of treatment versus the baseline values of the Schirmer’s test^(13)^. In 2016, Kawakita et al., studied the effect of oral pilocarpine administered for ≥3 months in patients with SS unresponsive to conservative treatment. They observed improvements in subjective eye symptoms, fluorescein staining scores, rose bengal staining scores, and tear film breakup time; nevertheless, there was no significant improvement noted in the values obtained from Schirmer’s testing^(19)^. The investigators concluded that, due to its efficacy and safety, oral pilocarpine is an option for the treatment of patients with severe dry eye. The variation in the Schirmer’s test observed in our study after the use of the drug was statistically significant. However, we do not believe that a change of a few millimeters in the tear flow could have been the main factor responsible for the improvement in the ocular surface observed through fluorescein and rose bengal staining. Similar to the acinus and ducts of the exocrine glands, the conjunctiva goblet cells also express muscarinic receptors on their surface and, therefore, are responsive to the action of cholinergic agonists (e.g., pilocarpine)^(20)^. It is possible that favorable changes induced in the morphology of epithelial cells of the ocular surface and in the number of goblet cells, as verified in previous studies, are also responsible for the improvement noted in the tear film breakup time^(13,21)^. In our study, we were able to confirm through both methods (i.e., the breakup time with fluorescein and the non-invasive breakup time) an improvement in tear stability after the administration of oral pilocarpine. We also observed improvement in the tear ferning patterns after treatment with pilocarpine, but not with placebo. This finding suggested that direct modifications on the ocular surface, in addition to the increase in tear flow, are responsible for the beneficial action of this medication. In the present study, improvement in the tear ferning patterns was certified by two independent examiners. Clearly, the phenomenon responsible for the formation of different tear ferning patterns is complex and depends on the interaction of different molecules. Kogbe et al., using electron microscopy, investigated the human tear ferning patterns. They concluded that the quantity and quality of the glycoproteins present in the tear samples are determinants for the formation of each specific pattern, as well as the concentration of electrolytes (particularly sodium, potassium, calcium, and magnesium)^(12,22)^. Although some previous studies have suggested that oral pilocarpine improves ocular dryness, this treatment modality is currently not popular among ophthalmologists, even in the treatment of the most severe cases. This may be attributed to the preference of ophthalmologists to prescribe topical medications. Moreover, the high incidence of systemic side effects associated with cholinergic drugs is an influencing factor. In the phase in which the drug was used, we observed that 96.8% of patients reported at least one side effect *versus* 56.2% with placebo. According to the results of our study, sweating was the most frequently reported side effect of pilocarpine (67.74%). Papas et al. studied 60 SS patients orally treated with 20 mg pilocarpine daily. They observed that sweating was present in 73%, whereas there were no serious clinical complications^(14)^. The absence of serious complications was also reported in a trial that included 373 patients with SS who received 20 mg of pilocarpine daily for the treatment of dry mouth, and were monitored clinically and through laboratory examinations^(23)^. Interestingly, in a study involving 40 patients with SS who received 15 mg of pilocarpine daily, the incidence of adverse effects was markedly lower (~20%)^(18)^. More recently, cevimeline (another cholinergic drug) was introduced for the oral treatment of dry mouth and possibly dry eyes of patients with SS. This agent is considered more selective, with no action on M4 receptors and possibly fewer side effects. However, due to its high cost and lack of availability in many countries, it is currently not popular. Pilocarpine is inexpensive and, therefore, accessible to most patients^(5,6)^. Moreover, its mechanism of action is different from those of all other options currently used for the treatment of dry eye.

Based on the present study, oral pilocarpine may be useful in the treatment of dry eye in patients with SS. Administration of 20 mg of pilocarpine daily for 10 weeks provided relief from signs and symptoms related to dryness and, consequently, improved the quality of life of patients. It was also clear that oral administration of this drug improves the ocular surface conditions, but does not return the ocular surface to its normal status. Therefore, we suggest that prescription of pilocarpine should be considered concurrently with other modalities for the treatment of dry eye. Future studies are warranted to investigate systemic treatment with pilocarpine for a longer period of time or in association with other drugs with different mechanisms of action (e.g., topical or systemic anti-inflammatory and immunomodulatory drugs).

## References

[1] Felberg S, Dantas PE. (2006). [Sjögren’s syndrome: diagnosis and
treatment]. Arq Bras Oftalmol.

[2] Fox RI, Stern M, Michelson P. (2000). Update in Sjögren syndrome. Curr Opin Rheumatol.

[3] Fox RI, Fox CM. (2016). Sjögren Syndrome: Why Do Clinical Trials
Fail?. Rheum Dis Clin North Am.

[4] (2007). Management and therapy of dry eye disease: report of the
Management and Therapy Subcommittee of the International Dry Eye WorkShop
(2007). Ocul Surf.

[5] Yamada H, Nakagawa Y, Wakamatsu E, Sumida T, Yamachika S, Nomura Y (2007). Efficacy prediction of cevimeline in patients with
Sjögren’s syndrome. Clin Rheumatol.

[6] Ono M, Takamura E, Shinozaki K, Tsumura T, Hamano T, Yagi Y (2004). Therapeutic effect of cevimeline on dry eye in patients with
Sjögren’s syndrome: a randomized, double-blind clinical
study. Am J Ophthalmol.

[7] Wiseman LR, Faulds D. (1995). Oral pilocarpine: a review of its pharmacological properties and
clinical potential in xerostomia. Drugs.

[8] Vitali C, Bombardieri S, Jonsson R, Moutsopoulos HM, Alexander EL, Carsons SE, European Study Group on Classification Criteria for Sjögren’s
Syndrome (2002). Classification criteria for Sjögren’s syndrome: a revised
version of the European criteria proposed by the American-European Consensus
Group. Ann Rheum Dis.

[9] Mangione CM, Lee PP, Gutierrez PR, Spritzer K, Berry S, Hays RD, National Eye Institute Visual Function Questionnaire Field Test
Investigators (2001). Development of the 25-item National Eye Institute Visual Function
Questionnaire. Arch Ophthalmol.

[10] Schiffman RM, Christianson MD, Jacobsen G, Hirsch JD, Reis BL. (2000). Reliability and validity of the Ocular Surface Disease
Index. Arch Ophthalmol.

[11] van Bijsterveld OP (1969). Diagnostic tests in the Sicca syndrome. Arch Ophthalmol.

[12] Rolando M. (1984). Tear mucus ferning test in normal and keratoconjuntivitis sicca
eyes. Chibret Int J Ophthamol;.

[13] Tsifetaki N, Kitsos G, Paschides CA, Alamanos Y, Eftaxias V, Voulgari PV (2003). Oral pilocarpine for the treatment of ocular symptoms in patients
with Sjögren’s syndrome: a randomised 12 week controlled
study. Ann Rheum Dis.

[14] Papas AS, Fernandez MM, Castano RA, Gallagher SC, Trivedi M, Shrotriya RC. (1998). Oral pilocarpine for symptomatic relief of dry mouth and dry eyes
in patients with Sjögrens syndrome. Adv Exp Med Biol.

[15] Papas AS, Sherrer YS, Charney M, Golden HE, Medsger TA Jr, Walsh BT (2004). Successful treatment of dry mouth and dry eye symptoms in
Sjögren’s syndrome patients with oral pilocarpine: A randomized,
placebo-controlled, dose-adjustment study. J Clin Rheumatol.

[16] Nelson JD, Friedlaender M, Yeatts RP, Yee R, McDermott M, Orlin S, The MGI PHARMA Sjögren’s Syndrome Study Group (1998). Oral pilocarpine for symptomatic relief of keratoconjunctivitis
sicca in patients with Sjögren’s syndrome. Adv Exp Med Biol.

[17] Takaya M, Ichikawa Y, Yamada C, Hoshina Y, Horiki T, Uchiyama M. (1997). [Treatment with pilocarpine hydrochloride for sicca symptoms in
Sjögren’s syndrome]. Ryumachi.

[18] Solans R, Bosch JA, Selva A, Simeón CP, Fonollosa V, Vilardell M. (2004). [Usefulness of oral pilocarpin therapy in the treatment of
xerostomia and xerophthalmia in patients with primary Sjögren’s
syndrome]. Med Clin (Barc).

[19] Kawakita T, Shimmura S, Tsubota K. (2015). Effect of oral pilocarpine in treating severe dry eye in patients
with Sjögren syndrome. Asia Pac J Ophthalmol (Phila).

[20] Fox RI, Konttinen Y, Fisher A. (2001). Use of muscarinic agonists in the treatment of Sjögren’s
syndrome. Clin Immunol.

[21] Aragona P, Di Pietro R, Spinella R, Mobrici M. (2006). Conjunctival epithelium improvement after systemic pilocarpine in
patients with Sjogren’s syndrome. Br J Ophthalmol.

[22] Kogbe O, Liotet S, Tiffany JM. (1991). Factors responsible for tear ferning. Cornea.

[23] Vivino FB, Al-Hashimi I, Khan Z, LeVeque FG, Salisbury PL 3rd, Tran-Johnson TK (1999). Pilocarpine tablets for the treatment of dry mouth and dry eye
symptoms in patients with Sjögren syndrome: a randomized,
placebo-controlled, fixed-dose, multicenter trial. P92-01 Study
Group. Arch Intern Med.

